# Glycosaminoglycan Storage Disorders: A Review

**DOI:** 10.1155/2012/471325

**Published:** 2011-10-05

**Authors:** Maria Francisca Coutinho, Lúcia Lacerda, Sandra Alves

**Affiliations:** ^1^Research and Development Unit, Department of Genetics, CGMJM, INSA, Portugal; ^2^Biochemical Genetics Unit, Department of Genetics, CGMJM, INSA, Portugal

## Abstract

Impaired degradation of glycosaminoglycans (GAGs) with consequent intralysosomal accumulation of undegraded products causes a group of lysosomal storage disorders known as mucopolysaccharidoses (MPSs). Characteristically, MPSs are recognized by increased excretion in urine of partially degraded GAGs which ultimately result in progressive cell, tissue, and organ dysfunction. There are eleven different enzymes involved in the stepwise degradation of GAGs. Deficiencies in each of those enzymes result in seven different MPSs, all sharing a series of clinical features, though in variable degrees. Usually MPS are characterized by a chronic and progressive course, with different degrees of severity. Typical symptoms include organomegaly, dysostosis multiplex, and coarse facies. Central nervous system, hearing, vision, and cardiovascular function may also be affected. Here, we provide an overview of the molecular basis, enzymatic defects, clinical manifestations, and diagnosis of each MPS, focusing also on the available animal models and describing potential perspectives of therapy for each one.

## 1. Introduction

The mucopolysaccharidoses (MPSs) are a group of lysosomal storage disorders caused by deficiency of enzymes catalyzing the stepwise degradation of glycosaminoglycans (GAGs) and characterized by intralysosomal accumulation and increased excretion in urine of partially degraded GAGs, which ultimately results in cell, tissue, and organ dysfunction [1]. 

Glycosaminoglycans (previously called mucopolysaccharides), with the exception of hyaluronic acid, are the degradation products of proteoglycans that exist in the extracellular matrix and are proteolytic cleaved, giving origin to GAGs, which enter the lysosome for intracellular digestion. There are four different pathways of lysosomal degradation of GAGs, depending on the molecule to be degraded: dermatan sulfate, heparan sulfate, keratan sulfate, and chondroitin sulfate. The stepwise degradation of glycosaminoglycans requires 10 different enzymes: four glycosidases, five sulfatases, and one nonhydrolytic transferase, whose structure, biosynthesis, processing, and cDNA sequence have already been extensively documented. Deficiencies of each one of these enzymes have already been reported and result in seven different MPSs, all of them sharing a series of clinical features, even though in variable degrees (summarized in Table 1) [1, 2]. 

Usually, MPSs are characterized by a chronic and progressive course, with different velocities of progression depending on the severity of each one. The typical symptoms include organomegaly, dysostosis multiplex, and a characteristic abnormal facies. Hearing, vision, and cardiovascular function may also be affected. Additionally, joint mobility may also be compromised. The majority of symptoms may be explained by abnormal accumulation of undegraded substrates within the lysosomes. In fact, the continued presentation of GAGs to cell for degradation results in storage, which gives rise to an enlargement of lysosomes. As substrates accumulate, the lysosomes swell and occupy more and more of the cytoplasm. As a consequence of this increased number and size of lysosomes, other cellular organelles may be obscured, and the nuclear outline may be deformed. As the process continues, the enlarged cells lead to organomegally. Abnormalities observed in heart cells and function may also be explained by GAGs accumulation. The increase of storage material within the cells of the heart valves causes an alteration of the cell's outline, changing them from fusiform to round. As a consequence, the valve leaflet and cordae tendinea become thickener and interfere with normal cardiac function, producing valvular stenosis. At corneal level, also, storage of undegraded GAGs results in reflection and refraction of light, leading to the cloudiness which is so typical of these pathologies. Also at the CNS level, swollen neurons and lysosomes may produce lesions that include the development of meganeurites and neurite sprouting (reviewed in [3, 4]).

Traditionally, MPSs are recognized through analysis of urinary GAGs. Several methods have been devised, to precise qualitative identification and quantitative measurements. These analyses of urinary GAGs allow discrimination between broad classes of MPSs but cannot distinguish subgroups. Definitive diagnosis is usually accessed through enzymatic assays of the defective enzyme in cultured fibroblasts, leukocytes, and serum or plasma (reviewed in [1]). During the last decade; however, dried blood spot technology was also introduced for enzymatic assays, allowing cheaper, easier, feasible diagnosis and opening the possibility for large population screenings (see Section 11 for more details).

In general, MPSs are transmitted in an autosomal recessive fashion, except for MPS II, which is X-linked. 

This paper provides an overview of the molecular basis, enzymatic defects, clinical manifestations, and diagnosis of each glycosaminoglycan storage disease, focusing also on the respective animal models and describing potential perspectives of therapy which are being tested as well as the ones which are already available (summarized in Table 2).

## 2. Mucopolysaccharidosis I

Mucopolysaccharidosis I is caused by a deficiency of *α*-L-iduronidase (IDUA; EC 3.2.1.76) and can result in a wide range of phenotypic involvement with three major recognized clinical entities: Hurler (MPS IH; MIM#607014), Hurler-Scheie (MPS IH/S; MIM#607015), and Scheie (MPS IS) syndromes. Hurler and Scheie syndromes represent phenotypes at the severe and mild ends of the MPS I clinical spectrum; respectively, and the Hurler-Scheie syndrome is intermediate in phenotypic expression [20]. It is important to stress that, although MPS I may be subdivided into these three clinically diverse entities, the underlying enzymatic defect is common to all of them, being all caused by mutation in the gene encoding *α*-L-iduronidase (*IDUA*).

Functionally, *α*-L-iduronidase is essential to the correct metabolism of both dermatan sulfate and of heparan sulfate, hydrolyzing the terminal *α*-L-iduronic acid residues of the above-referred glycosaminoglycans [1]. 

In 1992, Scott and colleagues [21] were able to clone and purify the gene that encodes this enzyme, *IDUA*, demonstrating that it spans approximately 19 kb and contains 14 exons. The first 2 exons are separated by an intron of 566 bp, a large intron of approximately 13 kb follows, and the last 12 exons are clustered within 4.5 kb. Previously, this gene was mapped to 4p16.3, through unequivocal in situ hybridization and southern blot analysis of mouse-human cell hybrids [22].

There are, presently, several animal models known for MPS I.

In 1979, Haskins and colleagues [5] described *α*-L-iduronidase deficiency in a cat, and, few years later, Shull et al. [6] and Spellacy et al. [23] reported a similar deficiency in the dog. Subsequent studies lead to cloning and characterization of the canine IDUA gene as well as the mutation causing the observed phenotype [24, 25] and proved it to be a good model for study of human MPS I. So, in 1994, Shull and collaborators [26] published the first results of enzyme replacement therapy in the canine model. Through intravenous administration of recombinant human *α*-L-iduronidase, these authors managed to obtain a remarkable resolution of lysosomal storage in both hepatocytes and Kupffer's cells. In the same year, Grosson et al. [27] mapped the homologous IDUA locus in the mouse to chromosome 5. That knowledge was later used to create a knock-out mouse presenting the characteristic MPS I features [7, 28].

Currently, both hematopoietic stem cell transplantation (HSCT) and enzyme replacement therapy (ERT) using laronidase (recombinant human *α*-L-iduronidase, Aldurazyme) are available for MPS I. HSCT is the recommended treatment for patients with severe MPS I, before 2 years of age [29–32]. ERT is recommended for the other cases, and it has been shown to be effective in ameliorating some of the clinical manifestations of MPS disease. Among positive effects are decreased hepatosplenomegaly, improved respiratory and myocardial function and physical capacity [33–35] as well as improvement in active movement followed by enhanced self-care [36]. Recently, several reports have been published trying to evaluate long-term effect of ERT on the natural history of treated patients. From those studies, several conclusions have been reached. Concerning treated patients' growth pattern, it became clear that children with MPS I grow considerably slower than healthy individuals, and differences between healthy and affected children increase with age [37]. Other relevant evidences show that early treatment of attenuated MPS I may significantly delay or prevent the onset of the major clinical signs, substantially modifying the natural history of the disease [38]. Additional investigation is needed to clarify the mechanisms by which improvements are achieved in laronidase-treated patients. Such knowledge may support the development of ERT directly targeting the brain.

### 2.1. Hurler's Syndrome (MPS IH)

Hurler's syndrome is the most severe form of MPS I and has been, over the last decades, the prototype description of MPS. Nevertheless, this may be misleading, since not all MPSs share the same features, and this pathology in particular is not representative of all of them, but only of the most severe end of a broad clinical spectrum (reviewed in [1]). Like all other MPSs, the clinical course of this disease is progressive, with multiple organ and tissue involvement. Hallmark clinical features of Hurler syndrome include coarse facies, corneal clouding, mental retardation, hernias, dysostosis multiplex, and hepatosplenomegaly. Children with Hurler's syndrome appear normal at birth and develop the characteristic appearance over the first years of life [39]. Length is often normal until about 2 years of age when growth stops; by age of 3 years, height is under the third percentile [40]. Cardiac disease and respiratory complications are common. Acute cardiomyopathy associated with endocardial fibroelastosis has been a presenting condition in some infants with MPS I less than 1 year of age [41]. Upper and lower respiratory tract infections are also frequent [42]. Developmental delay is often apparent by 12 to 24 months of age, with a maximum functional age of 2 to 4 years followed by progressive deterioration. Most children develop limited language as a consequence of developmental delay, chronic hearing loss, and enlarged tongue [1]. Dermal melanocytosis may also be found in Hurler patients [43], as well as in patients suffering from other LSDs, such as GM1 gangliosidosis. Nevertheless, Hurler's syndrome is the most common lysosomal storage disease associated with dermal melanocytosis, as revealed by a literature analysis.

### 2.2. Hurler-Scheie's Syndrome (MPS IH/S)

MPS IH/S corresponds to a clinical phenotype which is intermediate between the Hurler and the Scheie syndromes. It is characterized by progressive somatic involvement with dysostosis multiplex but little or no mental retardation. First symptoms usually occur between 3 and 8 years. Characteristic features of Hurler's syndrome, such as corneal clouding, joint stiffness, deafness, and valvular heart disease, can also appear in MPS IH/S patients. Nevertheless, the onset of these symptoms occurs much later than that in the severe MPS I type, beginning in the midteens and leading to significant impairment and loss of function. Other clinical features, such as micrognathism, pachymeningitis cervicalis, and compression of the cervical cord due to GAG accumulation in the dura, may also occur. Cardiac and respiratory complications may explain the high clinical mortality (reviewed in [1]).

### 2.3. Scheie's Syndrome (MPS IS)

Scheie's syndrome was earlier thought to be a separate entity designated MPS V, instead of a phenotypical subtype of MPS I [44]. This pathology is characterized by a mild phenotype in which dysostosis multiplex can be present. Joint involvement is marked in the hand with a claw-hand deformity. Patients also have genu valgum, stiff, painful feet, and *pes cavus* [1]. Cardiac and respiratory complications are much milder than in the Hurler syndrome, with aortic and mitral valvular disease being a common feature [45]. At a respiratory level, Perks et al. [46] have reported two brothers with Scheie's syndrome suffering from sleep apnea, but no other complications are known. Intelligence is normal [1]. Pachymeningitis cervicalis (compression of the cervical cord secondary to glycosaminoglycan accumulation in the dura) may also occur.

## 3. Mucopolysaccharidosis II (Hunter's Syndrome)

Mucopolysaccharidosis II is the sole MPS transmitted in an X-linked manner and is caused by deficiency of the lysosomal enzyme iduronate sulfatase, which is crucial to the correct degradation of heparan and dermatan sulfate, by cleaving their O-linked sulfate. As a result, there is a progressive accumulation of glycosaminoglycans in nearly all cell types, tissues, and organs. Patients with MPS II excrete excessive amounts of dermatan sulfate and heparan sulfate in the urine [20, 47]. Hunter syndrome is caused by mutation in the gene encoding iduronate-2-sulfatase (*IDS*).

Although the disease is known since the early 1970s, being the first MPS to be defined clinically in humans, it was not until the 1990s that the IDS was cloned. In 1991, Wilson et al. [48] localized the gene to Xq28. Two years later, Flomen and coworkers [49] described the gene's structure as containing 9 exons and characterized the intron sequences surrounding them. In the same year, Wilson et al. [50] reported the complete sequence of the *IDS *gene, which spans approximately 24 kb. The potential promoter for *IDS* lacks a TATA box but contains GC box consensus sequences, which are consistent with its role as a housekeeping gene.

Curiously, a second *IDS* gene (*IDS2*) was identified by Bondeson et al. [51]. It is a pseudogene and is located within 90 kb telomeric region of the *IDS* gene and involved in a recombination event with the primary *IDS* gene in about 13% of patients with the Hunter syndrome.

Traditionally, the Hunter syndrome comprises 2 recognized clinical entities, according to the severity of symptoms: mild and severe. Although largely used, this nomenclature does have its difficulties, since the mild and severe forms represent the two ends of a wide and continuous spectrum of clinical severity. Also, in terms of iduronate deficiency, these forms cannot be distinguished since the enzyme's activity is equally deficient in both (reviewed in [1]). They are, though, separated almost exclusively on clinical grounds, although nowadays mutation analysis may help distinguish them. 

This classification of MPS goes back to 1972, when McKusick distinguished between the severe form (which he called MPS IIA), with progressive mental retardation and physical disability and death before age 15 years in most cases, and the mild form (called MPS IIB) compatible with survival to adulthood and in which intellect is impaired minimally, if at all. He also pointed out the lack of corneal clouding in the X-linked form of MPS as opposed to the autosomal forms.

Presently, this classification has become obsolete since, in 2008, Wraith et al. [47] stated that MPS II should be regarded as a continuum between the two extremes (severe and attenuated). They noted that, although the clinical course for the more severely affected patients is relatively predictable, there is considerable variability in the clinical phenotype and progression of the more attenuated form of the disease and, so, it would not be correct to consider the milder form as a separate entity but, instead, look at Hunter's disease as a phenotypical continuum, with several possible degrees of severity.

In 1998, Wilkerson et al. [8] described Hunter's syndrome in a Labrador retriever, with the typical clinical features observed in humans: coarse facies, macrodactyly, corneal dystrophy, progressive CNS deterioration, and positive biochemical diagnosis for MPS through urine analysis.

After the successful results obtained in improving certain disease manifestations in patients with MPS I, including visceral manifestations and attenuation of neurologic disease progression [29, 52], hematopoietic stem cells transplantation (HSCT) has also been performed in several patients with MPS II. Unfortunately, although the transplantation of hematopoietic stem cells provides some enzymatic reconstruction in many target tissues with decreased excretion of GAGs in urine, decreased liver and spleen volumes, diminished facial coarsening, and improved respiratory function and joint mobility [53, 54], the results at neurological level were disappointing (reviewed in [55]). The additional risk of morbidity and mortality associated to this procedure led investigators to focus their attention in ERT for this pathology, with much better results, as discussed below. 

A knock-out mouse model for MPS II was developed by replacing exon 4 and a portion of exon 5 of *IDS* with the neomycin-resistance gene [9, 56]. Affected mice exhibit a phenotype with notorious similarities to human disease, both at the biochemical and the clinical levels [9]. Several studies with this knock-out mouse model were done to assess the effect of ERT [56] as well as dose and various dosing regimens of idursulfase in urine and tissue GAG levels [57]. The results of these studies were quite promising, with a marked decrease in urinary GAGs as well as decreased GAG accumulation in several tissues [56] verified for several idursulfase doses and several dosing frequencies [57]. These studies have been used to support the first clinical trial of recombinant *IDS* in Hunter's syndrome patients. At the moment, both phase I/II [58] and phase II/III [59] clinical studies have proven not only the efficacy but also the safety of idursulfase replacement therapy. Consequently, ERT with recombinant human iduronate sulfatase (Elaprase, idursulfase, Shire Human Genetic Therapies Inc.) was approved in the US (July, 2006) and the European Union (January, 2007) for the treatment and the management of MPS II. The recommended dose is 0.5 mg/kg administered once weekly as an intravenous infusion (reviewed in [55]). As time goes by, additional evidence on the efficacy of ERT for MPS II patients is being published, as long-term treatments are successful. This is the case of a recently published report on the improvements observed in a 7 years and 10 months old child who began a 36 months' treatment with Elaprase at 4 years and 10 months. At the end of the treatment, the child presented normal excretion of GAGs in urine, normal-sized liver and spleen, and significant bone remodeling. Cardiac and neurological development, however, still progressively deteriorated [60]. This year, protective effects of ERT in MPS II patients were also reported for DNA damaging in leukocytes [61] and oxidative stress [62].

## 4. Mucopolysaccharidosis III (Sanfilippo's Syndrome)

The Sanfilippo syndrome, or mucopolysaccharidosis III, is caused by impaired degradation of heparan sulfate [1] and includes 4 subtypes, each due to the deficiency of a different enzyme: heparan N-sulfatase (type A; MIM no. 252900), *α*-N-acetylglucosaminidase (type B; MIM no. 252920), acetyl CoA: *α*-glucosaminide acetyltransferase (type C; MIM no. 252930), and N-acetylglucosamine-6-sulfatase (type D; MIM#252940). At a clinical level, the four subtypes are quite similar, with a characteristic severe central nervous system degeneration associated with mild somatic disease. Onset of clinical features usually occurs between 2 and 6 years, severe neurologic degeneration occurs in most patients between 6 and 10 years of age, and death occurs typically during the second or third decade of life. Type A has been reported to be the most severe, with earlier onset and rapid progression of symptoms and shorter survival [63].

### 4.1. Mucopolysaccharidosis IIIA (Sanfilippo A)

General MPS IIIA clinical features include severe mental retardation with relatively mild somatic features (moderately severe claw hand and visceromegaly, little or no corneal clouding, little or no vertebral change). Usually, this pathology is characterized by marked overactivity, destructive tendencies, and other behavioral aberrations. 

MPS IIIA phenotype is caused by mutations in the gene encoding N-sulfoglucosamine sulfohydrolase, also named heparan sulfate sulfatase (*SGSH*; 605270). This enzyme is specific for sulfate groups linked to the amino group of glucosamine.

In 1995, the gene encoding N-sulfoglucosamine sulfohydrolase, *SGSH*, was isolated, sequenced, and cloned [64]. Later, it was shown to contain 8 exons spanning approximately 11 kb [65].

There are two animal models known for MPS IIIA. The first to be discovered was the canine model when Fischer et al. [10] identified sulfaminidase deficiency in two adult wire-haired dachshund littermates. Subsequently, Aronovich et al. [66] determined the normal sequence of the canine heparan sulfate sulfatase gene and cDNA, through PCR-based approaches. Another model was described in 2001, when Bhattacharyya and collaborators [11] found a spontaneous mouse mutant of MPS IIIA resulting from a missense mutation (D31N) in the murine sulfatase gene. Affected mice die at about 10 months of age, exhibiting notorious visceromegaly, distended lysosomes and heparan sulfate accumulation in urine. Hemsley and Hopwood [67] found that these mice had severe brain involvement, with impaired open field locomotor activity and behavioral changes, suggesting axonal degeneration. Later, Settembre et al. [68] observed increased autophagosomes resulting from autophagosome-lysosome function in these mice. Similar findings were observed in another mouse model of another lysosomal storage disorder (multiple sulfatase deficiency; MSD; MIM no. 272200), reinforcing the recent idea that these diseases are disorders of autophagy, which may be a common mechanism for neurodegenerative lysosomal storage disorders.

MPS IIIA mice were recently tested for substrate deprivation therapy with both genistein and rhodamine B, two chemicals that inhibit GAG synthesis ([4, 69], reviewed in [70]). Encouraging results were obtained with both compounds, and this therapeutic approach started to be considered for several MPSs (see Section 11 for more details). Other interesting results were also obtained when siRNAs were used to reduce GAG synthesis in MPS IIIA mice. Last year, this approach was tested by Dziedzic et al. [71], who managed to reduce mRNA levels of four genes, *XYLT1, XYLT2, GALTI*, and *GALTII*, whose products are involved in GAG synthesis. This decrease of levels of transcripts corresponded to a decrease in levels of proteins encoded by them. Moreover, efficiency of GAG production in these fibroblasts was considerably reduced after treatment of the cells with siRNA. Either way, substrate deprivation therapy seems to be a promising approach for Sanfilippo's syndrome type A.

Gene therapy approaches are also being tested in MPS IIIA mice. Recently, promising results have been reported by Fraldi et al. [72], who performed experiments with intracerebral adeno-associated-virus- (AAV-) mediated delivery of *SGSH* gene, together with *SMUF1 *gene, which exhibits an enhancing effect on sulfatase activity when coexpressed with sulfatases. They observed a visible reduction in lysosomal storage and inflammatory markers in transduced brain regions, together with an improvement in both motor and cognitive functions.

### 4.2. Mucopolysaccharidosis IIIB (Sanfilippo B)

With a phenotype quite similar to MPS IIIA, the Sanfilippo syndrome B is characterized by deficiencies of *α*-N-acetylglucosaminidase, caused by mutations in the *NAGLU* gene that encodes this enzyme. *α*-N-Acetylglucosaminidase is required for the removal of the N-acetylglucosamine residues that exist in heparan sulfate or are generated during lysosomal degradation of this polymer by the action of heparan acetyl-CoA: *α*-glucosaminide N-acetyltransferase (reviewed in [1]).

The *NAGLU *gene was cloned in 1995 by Zhao and colleagues [73]. The deduced 743-amino acid protein has a 20- to 23-residue leader sequence, consistent with a signal peptide, and 6 potential N-glycosylation sites. It contains 6 exons and spans 8.3 kb on chromosome 17q21 [74].

Similarly to the above-referred MPS III syndrome, there is also a natural occurring mutant for Sanfilippo B. It was described by Ellinwood and coworkers, in 2003, in Schipperke's dogs [12]. 

During the last decade, Li et al. [75] created a laboratorial murine MPS IIIB was also constructed trough targeted disruption of the *NAGLU* gene [76]. With a phenotype quite similar to that of patients with MPS IIIB, this model began immediately to be used for therapeutic approaches as well as for pathogenesis studies. The first studies were done to evaluate the potential of ERT for this pathology [76]. The results, however, were quite disappointing since the recombinant NAGLU produced in Chinese hamster ovary (CHO) cells was not efficiently captured by MPS IIIB cells, either *in vitro* [77, 78] or *in vivo* [76]. This difficulty has turned the search for a treatment for MPS IIIB even more challenging. Presently, several therapies are under evaluation for this disease, including cell-mediated therapy, enzyme enhancement therapy, substrate deprivation therapy, and viral gene therapy (reviewed in [79]).

Promising results are being achieved through gene therapy approaches in MPS IIIB mice, namely, through direct microinjection into the brain of adeno-associated virus (AAV) vectors coding for NAGLU [80–82] and intravenous injections and intracranial gene delivery of lentiviral (LV) vector of NAGLU [83–85].

### 4.3. Mucopolysaccharidosis IIIC (Sanfilippo C)

Sanfilippo syndrome C is, in general, characterized by the same clinical features described to MPS IIIA. Nevertheless, the enzyme deficiency in this pathology is different from the one causing the latter. Type C disease is caused by mutations in the gene encoding heparan acetyl-CoA: *α*-glucosaminide N-acetyltransferase (*HGSNAT*; 610453). This is the only known lysosomal enzyme that is not a hydrolase. It catalyzes the acetylation of the glucosamine amino groups that have become exposed by the action of heparan-N-sulfatase (reviewed in [1]).

The *HGSNAT* gene was cloned in parallel by two different groups, during the last decade: Fan et al. [86] and Hřebíček et al. [87]. The molecular defects underlying MPS IIIC remained unknown for almost three decades due to the low tissue content and the instability of *HGSNAT *[88].

To date, 54 *HGSNAT* sequence variants have been identified including 13 splice-site mutations, 11 insertions and deletions with consequent frameshifts and premature termination of translation, 8 nonsense, and 18 missense (reviewed in [89]).

Recently, two independent studies from Feldhammer et al. [88] and Fedele and Hopwood [90] have performed exhaustive functional analysis of the majority of the missense mutations already reported for the *HGSNAT* gene. Attention was focused in this particular type of mutations since there are several MPS IIIC patients carrying only missense mutations, either homozygous or heterozygous, who present an unexpected severe phenotype. In fact, although splicing and frameshift mutations are usually associated to that type of phenotype, since they give rise to premature termination codons and trigger nonsense-mediated mRNA decay (NMD); missense mutations are traditionally associated to milder disease. Nevertheless, this typical/general pattern is not observed for MPS IIIC. That is why these alterations were specifically cloned, expressed, and analyzed for their folding, targeting, and enzymatic activities. As a result, Fedele and Hopwood [90] have observed that the expression levels and enzymatic activity of most mutants were extremely low or even negligible. Feldhammer and colleagues [88], on the other hand, have observed that those mutations cause a misfolding of the enzyme, which is not correctly glycosylated. As a consequence, HGSNAT is not targeted to the lysosome but, instead, stays in the endoplasmic reticulum (ER). Thus, enzyme folding defects due to missense mutations, together with NMD seem to be the major molecular mechanisms underlying MPS IIIC. This makes MPS IIIC a good candidate for enzyme enhancement therapy, where active site-specific inhibitors are used as pharmacological chaperones to modify the conformation of the mutant lysosomal enzymes usually retained and degraded in the ER, in order to increase the level of the residual activity to a point which is sufficient to reverse the clinical phenotypes [88]. Together with inhibitors of heparan sulphate synthesis, pharmacological chaperones are currently being tested to reduce storage of this polymer in the CNS to levels sufficient to stop neuronal death and reverse inflammation.

### 4.4. Mucopolysaccharidosis IIID (Sanfilippo D)

Like the previous MPS III subtypes, Sanfilippo's syndrome D presents a phenotype similar to MPS IIIA, with a singular enzyme deficiency underlying it: mutation in the gene encoding N-acetylglucosamine-6-sulfatase (GNS; 607664). The enzyme was originally described as specific for the 6-sulphated N-acetylglucosamine residues of heparan sulphate. However, the early data have been reinterpreted, and given that this sulfatase is in fact able to desulphate the 6-sulphated N-acetylglucosamine present in *α*- or in *β*-linkage or even as a free monosaccharide (reviewed in [1]).

N-Acetylglucosamine-6-sulfatase (EC 3.1.6.14) was purified and characterized by Freeman et al. [91], who identified 4 different forms of the enzyme in liver. Its catalytic properties were studied by Freeman and Hopwood [92]. Afterwards, Robertson et al. [93] assigned the glucosamine-6-sulfatase gene, which they symbolized *G6S*, to chromosome 12q14 by *in situ* hybridization of a tritium-labeled *G6S* cDNA probe. The localization was confirmed by using the cDNA clone in analyses of DNA from human/mouse hybrid cell lines. More recently, that information was completed by the work of Mok et al. [94], who amplified and sequenced the promoter and 14 exons of the *GNS* gene from a patient with MPS IIID. By analyzing that patient, it was also possible to identify a homozygous nonsense mutation in exon 9, predicted to result in premature termination at codon 355, as well as two common synonymous coding SNPs. At the same time, another group identified a 1-bp deletion in the *GNS* gene in another affected individual [95].

A naturally occurring large animal model was described by Thompson et al. [13], who reported type D Sanfilippo's syndrome in a Nubian goat. Later, caprine MPS IIID was used to evaluate the efficacy of ERT in this pathology. Recombinant caprine N-acetylglucosamine-6-sulfatase was administered intravenously to one MPS IIID goat at 2, 3, and 4 weeks of age. As a result, a marked reduction of lysosomal storage vacuoles was observed in hepatic cells, but no amelioration was noticed concerning the CNS lesions. No residual enzyme activity was observed either in brain or liver. Taking this preliminary results into account, it was considered that other treatment regimens will be necessary for MPS IIID [96].

## 5. Mucopolysaccharidosis IV

Mucopolysaccharidosis IV, or Morquio's syndrome, is caused by impaired degradation of keratan sulphate. Presently, there are two known enzyme deficiencies causing 2 different subtypes of Morquio's syndrome: deficiency in N-acetylglucosamine-6-sulfatase (causing Morquio's disease type A; MIM no. 253000) and deficiency in *β*-galactosidase (causing Morquio's disease type B; MIM no. 253010). Both MPS IV subtypes present a wide spectrum of clinical manifestations, but there are some characteristic common features: short trunk dwarfism, fine corneal deposits, spondyloepiphyseal dysplasia. Actually, the predominant clinical features of Morquio's syndrome are the ones related to the skeleton. Most of the times, this severe somatic disease is accompanied by a normal intelligence [1]. Patients with the severe phenotype do not normally survive past the second or third decade of life [97].

### 5.1. Morquio's Syndrome Type A

Morquio's syndrome A is caused by mutations in the gene encoding galactosamine-6-sulphate sulfatase (*GALNS*), which plays a crucial role on the degradation of both keratan sulphate and chondroitin sulphate.

The gene coding for human galactosamine-6-sulphate sulfatase (*GALNS*), was mapped to chromosome 16q24.3 trough fluorescence *in situ* hybridization assays [98]. Its structure was described at the same time by independent groups as comprising 14 exons and spanning approximately 40–50 kb [99, 100]. Curiously, the *GALNS* gene contains an *Alu* repeat in intron 5 and a VNTR-like sequence in intron 6 [100].

No natural occurring model is known for either type A or type B Morquio's syndrome. Nevertheless, a laboratorial murine model for type A syndrome was created from an induced disruption in exon 2 of the *GALNS* gene. Mutants presented no detectable enzyme activity and showed increased GAG levels in urine. GAGs accumulation was also detected in several tissues including liver, kidney, spleen, heart, brain, and bone marrow [14]. These mice were later tested for enzyme replacement therapy and, after a 12-week long treatment with native GALNS or SUMF1-modified GALNS, showed manifest clinical improvement, demonstrated by a marked reduction of storage material in visceral organs, bone marrow, heart valves, ligaments, and connective tissue. The clearance of stored material in brain was dose dependent, and the keratan sulphate blood levels were reduced to normal [101]. 

Presently, there is no effective therapy for MPS IVA and care has been palliative, as in the majority of LSDs. Enzyme replacement therapy (ERT) and hematopoietic stem cells therapy (HSCT) have been considered as potential therapeutic approaches for MPS IVA (reviewed in [102]), ERT being, though, the most attractive candidate, since affected patients lack CNS involvement.

Recently, Rodríguez et al. [103] have produced a recombinant GALNS enzyme in *Escherichia coli* BL21. To produce sufficient amounts of purified GALNS enzyme, high level expression of GALNS in Chinese hamster ovary (CHO) cells has been established as a source of selectively secreted human recombinant enzyme. This recombinant enzyme has already been tested in the murine knock-out model, with consequent clearance of tissue and blood keratan sulphate [101]. These results provided important preclinical data for the design of GALNS ERT trials, which are now in course.

### 5.2. Morquio's Syndrome Type B

Although presenting overlapping clinical features, Morquio's syndrome B is genetically distinct from Morquio's syndrome A, being caused by impairments in another enzyme involved in the stepwise degradation of keratan sulphate: *β*-galactosidase, which is coded by the *GLB1* gene. Beta galactosidase hydrolases terminal *β*-linked galactose residues found in GM1 ganglioside, glycoproteins, and oligosaccharides, as well as in keratan sulphate (reviewed in [1]).

The *GLB1* gene spans 62.5 kb and contains 16 exons [104, 105] and maps to chromosome 3p21.33 [106]. The deduced 677-residue protein has a calculated molecular mass of 75 kD and contains a putative 23-residue signal sequence and 7 potential asparagine-linked glycosylation sites. It may be interesting to refer that the *GLB1* gene gives rise to 2 alternatively spliced mRNAs: a major 2.5-kb transcript that encodes the classic lysosomal form of the enzyme of 677 amino acids, and a minor 2.0-kb transcript that encodes a *β*-galactosidase-related protein (elastin-binding protein, EBP) of 546 amino acids with no enzymatic activity and a different subcellular localization. Exons 3, 4, and 6 are absent in the 2.0-kb mRNA as a consequence of alternative splicing of the pre-mRNA [107–109]. 

Presently, there are no known animal models for MPS IVB, either natural or engineered.

## 6. Mucopolysaccharidosis V

The designation MPS V is no longer used. In fact, the phenotype which was first classified as MPS V, was later found to be the milder form of MPS I (Scheie's syndrome), caused by deficiencies in *α*-L-iduronidase, with the typical stiff joints, clouding of the cornea most dense peripherally, survival to a late age with little if any impairment of intellect and aortic regurgitation [44].

## 7. Mucopolysaccharidosis VI (Maroteaux-Lamy Syndrome)

Mucopolysaccharidosis type VI is an autosomal recessive lysosomal storage disorder resulting from a deficiency of arylsulfatase B (N-acetylgalactosamine-4-sulfatase). Clinical features and severity are variable but usually include short stature, hepatosplenomegaly, dysostosis multiplex, stiff joints, corneal clouding, cardiac abnormalities, and facial dysmorphism. Intelligence is usually normal [110].

Arylsulfatase B is a lysosomal enzyme that removes the C4 sulphate ester group from the N-acetylgalactosamine sugar residue at the nonreducing terminus of dermatan sulphate and chondroitin sulphate, during lysosomal degradation [111]. The gene that codes for this enzyme was first mapped to chromosome 5q11-q13 [112] and is now known to contain 8 exons and span about 206 kb [111].

In 2002, a 3-year-old Siamese/short-haired European cat was referred for clinical disease characterized by dwarfism, facial dysmorphia, paralysis, small and curled ears, corneal clouding, and large areas of alopecia. X-ray examination showed multiple bone dysplasias. These features lead to suspect from a mucopolysaccharide storage disorder. Subsequent analysis proved it to be a natural occurring form of the Maroteaux-Lamy syndrome [15].

This MPS VI model has been extensively used over the last years to test ERT for this specific pathology. In 2003, Auclair and colleagues [113] have evaluated the cats' response to infusions of recombinant human N-acetylgalactosamine-4-sulfatase (rhASB) and observed an overall improvement in the disease condition at physical, neurological, and skeletal levels. Later, the same team has demonstrated that a high rate of immunotolerance towards rhASB can be achieved in MPS VI cats with a short-course tolerisation regimen [114], which may help the implementation of such procedures. Another interesting approach was designed, specifically to ameliorate joint disease in MPS IVA, through long-term articular administration of rhASB, leading to a notorious improvement in feline joint disease [115]. These successful results lead to the development of clinical trials in MPS VI patients, and three clinical studies including 56 patients have evaluated the efficacy and safety. As a consequence, enzyme replacement therapy (ERT) became available. The specific ERT for MPS VI, galsulfase (Naglazyme, Biomarin Pharmaceutical) was approved in 2005 by FDA and in 2006 by EMA. Long-term follow-up data with patients treated up to 5 years showed that ERT is well tolerated and associated with sustained improvements in the patients' clinical condition [2, 116]. 

Even though presently there is ERT available for these patients, other therapeutic approaches are being tested in animal models for MPS VI. In 2009, the first attempt of successful gene therapy was performed through lentiviral-mediated gene transfer to joint tissues of the rat, with consequent correction of MPS VI cells [117]. This year, another study, involving intravascular administration of adeno-associated viral vectors in MPS VI cats, was published. After gene transfer the authors observed clearance of GAG storage, improvement of long bone length, reduction of heart valve thickness, and improvement in spontaneous mobility [118]. Either way, promising therapeutic strategies for MPS VI patients may be arising.

## 8. Mucopolysaccharidosis VII (Sly's Syndrome)

MPS VII, also known as Sly's syndrome, is characterized by the impossibility to degrade glucuronic acid-containing GAGs, due to impaired function of *β*-glucuronidase, which removes the glucuronic acid residues present in dermatan sulphate as well as in heparan and chonroitin sulphates (reviewed in [1]). Clinical features are highly variable, with phenotypes ranging from severe fetal hydrops to mild forms allowing survival into adulthood. Typical features include hepatomegaly, skeletal abnormalities, coarse facial features, and variable degrees of mental impairment [119].

MPS VII was first reported by Sly and collaborators in 1973, in a boy with skeletal changes consistent with MPS, hepatosplenomegaly, and granular inclusions in granulocytes. Additional features included hernias, unusual facies, protruding sternum, thoracolumbar gibbus, vertebral deformities, and mental deficiency. When *β*-glucuronidase activity was measured in fibroblasts, obtained values were less than 2% of control values. Both parents and several sibs of the mother showed an intermediate level of the enzyme [120].

In 1990, Miller et al. [121] reported that the gene encoding *β*-glucuronidase (*GUSB)* is 21 kb long, contains 12 exons, and gives rise to two different types of cDNAs, through an alternate splicing mechanism. Speleman et al. [122] used fluorescence in situ hybridization to map the *GUSB* gene to 7q11.21-q11.22. This map position was confirmed by dual-color hybridization of *β*-glucuronidase and another gene which had been mapped proximal to it: elastin (7q11.23).

Several pseudogenes, located on chromosomes 5, 6, 7, 20, 22, and Y, were also detected by Shipley et al. [123], when amplifying exons 2–4, 3, 6-7, and 11. 

In 2009, Tomatsu et al. [124] provided a review of mutations in the *GUSB *gene that cause MPS type VII. Forty-nine different pathogenic mutations have been reported in the literature, with approximately 90% of them being missense mutations. Approximately 40% of the known *GUSB* mutations occur at CpG sites within the gene. The most common mutation is L176F, which has been found in several populations: American (Caucasian), Brazilian, British, Chilean, French, Mexican, Polish, Spanish, and Turkish ([125–127], reviewed in [124]). Genotype/phenotype analysis indicated that the most severe phenotype was associated with truncating mutations and with mutations affecting either the hydrophobic core or the modification of packing.

In 1984, mucopolysaccharidosis type VII (Sly syndrome) was described in a mixed-breed dog [16]. Since then, several other affected dogs have been studied, in the animal colony established at the University of Pennsylvania, the School of Veterinary Medicine [128] and, later, in a 12-week-old male German Shepherd dog studied in the same school [129]. All dogs shared the same missense mutation and developed similar phenotypes with skeletal deformities, corneal cloudiness, cytoplasmic granules in the neutrophils and lymphocytes of blood and CSF, and glycosaminoglycans in urine [129]. Another animal model was described as naturally occurring: the gus^mps/mps^ mouse, which has a 1 bp deletion in exon 10 resulting in a progressive degenerative disease that reduces lifespan and causes facial dysmorphism, growth retardation, deafness, and behavioral defects [17]. Nevertheless, opportunities for experimental therapies were greatly expanded by the work of Tomatsu et al., in 2006 [18], who developed a new MPS VII mouse model, which is tolerant to both human and murine GUS, without the characteristic immune responses that complicated evaluation of the long-term benefits of enzyme replacement or gene therapy when the naturally occurring mice were used. Ever since, several therapeutic approaches have been attempted in MPS VII mice, and the results have been encouraging. That is the case of the works by Bosch and collaborators, who have been working on gene therapy for this pathology, in order to correct brain lesions. They have used both adeno-associated virus (AAV) [130] and lentivirus-mediated gene transfer [131] and observed that there was a significant correction of pathology in the brain of affected mice.

Other therapeutic approaches had already been attempted, but their results were not as promising. In fact, in 1998, allogenic bone marrow transplantation was reported in a 12-year-old Japanese girl with consequent improvement of motor function and daily life activities, decrease of upper respiratory and ear infections, but no improvement at all in cognitive function [132].

## 9. Mucopolysaccharidosis VIII

The clinical entity once known as MPS VIII was described in a single patient, in the late 1970s. The patient, a 5-year-old child, presented short stature, coarse hair, hepatomegaly, mild dysostosis multiplex, mental retardation, and no signs of corneal clouding. Biochemical analysis of the urine revealed increased excretion of keratan and heparan sulphate [133, 134]. The biochemical findings described by this group lead to suspect the existence of two hexosamine sulfatases and propose the existence of this novel MPS, caused by glucosamine-6-sulfatase [133]. 

Nevertheless, subsequent analysis on Diferrante's laboratory brought this idea down, and the designation MPS VIII was abandoned [135].

## 10. Mucopolysaccharidosis IX

Mucopolysaccharidosis IX, also known as hyaluronidase deficiency, is caused by mutations in the *HYAL1* gene.

This disease was first discovered by Natowicz et al. [136] in a 14-year-old girl with short stature and multiple periarticular soft-tissue masses. Radiographic analysis showed nodular synovia, acetabular erosions, and a popliteal cyst. Lysosomal storage of hyaluronan (HA) was evident within the macrophages and fibroblasts of biopsied soft-tissue masses, and serum concentrations were elevated 38–90-fold. She was proven to have a storage disease of hyaluronan (hyaluronic acid) due to a genetic deficiency of hyaluronidase. The descriptions of hyaluronidase deficiency in this family are consistent with autosomal recessive inheritance.

In order to determine the molecular basis of MPS IX, Triggs-Raine et al. [137] analyzed 2 different candidate genes tandemly distributed on chromosome 3p21.3, both encoding proteins with homology to a sperm enzyme with hyaluronidase activity. These genes, *HYAL1* and *HYAL2*, encode 2 distinct lysosomal hyaluronidases with different substrate specificities. When characterizing the patient with hyaluronidase deficiency originally reported in [136], they verified that he was a compound heterozygote for 2 mutations in the *HYAL1* gene: a missense mutation (c.1412G>A), which introduced a nonconservative amino acid substitution in a putative active site residue (p.Glu268Lys) and a complex intragenic rearrangement, 1361del37ins14, which resulted in a premature termination codon. Through this work, they have also showed that these 2 hyaluronidase genes, together with a third adjacent HYAL3 gene, had markedly different tissue expression patterns, consistent with differing roles in the metabolism of hyaluronan. These findings allowed this team not only to explain the unexpectedly mild phenotype of MPS IX but also to predict the existence of other hyaluronidase-deficiency disorders.

Presently, three other hyaluronidase-related genes (*HYAL4*, *HYALP1*, *SPAM1*) have been identified at 7q31.3 [138]. These genes are predicted to encode hyaluronidases, endoglycosidases that initiate the degradation of HA, a large negatively charged GAG found in the extracellular matrix (ECM) of all vertebrate cells [19]. 

Since there is only one patient reported to date, the development and characterization of a model of Hyal1 deficiency was the first logical step in understanding the main phenotypic symptoms associated with MPS IX. During this decade, a mouse model for MPS IX has become available and was fully characterized [19]. Overall, it was observed that the murine MPS IX model displays the key features of the human disease. Nevertheless, during the same year, another mutant mouse suffering from a hyaluronidase deficiency was described, this one deficient in HYAL2 [139]. Skeletal and hematological anomalies were described in this model, raising the possibility that a similar defect, defining a new MPS disorder, exists in humans [139].

## 11. Conclusion

The elucidation of enzyme deficiencies underlying mucopolysaccharidoses was crucial to unveil the normal pathways of glycosaminoglycan catabolism. In fact, only through the consequences of their absence became the role of several enzymes evident. The majority of these enzyme deficiencies were discovered during the 1970s. Over the last decades the enzyme deficiencies underlying each disease, and the molecular defects causing them have been identified and extensively analyzed and characterized. As a result, six different MPS are known, caused by deficiencies in one of the ten different enzymes necessary to intralysosomal degradation of GAGs through one of the four different degradation pathways.

Each disease has its own hallmark features. Nevertheless, a common pattern arouses: MPS are usually chronic, with a progressive course and different severity degrees. Organomegaly, dysostosis multiplex, and CNS involvement are common but not necessary features.

Over the years, several MPS have been recognized in animals as naturally occurring diseases, and others were created by knock-out technology. Most animal colonies have been established from single related heterozygous animals, in such a way that the affected offspring is homozygous for the same mutant allele. All these models present disease pathology similar to that seen in humans, making the animals extremely valuable for both investigation of disease pathogenesis and testing of therapies. Large animal homologues are similar to humans in natural genetic diversity, approaches to therapy and care, and possibility of evaluating long-term effects of treatment. Presently, therapeutic strategies for MPS include enzyme replacement therapy, heterologous bone marrow transplantation, and somatic cell gene transfer, all of which have been tested in animals with some success. During the 80s, transplantation of hematopoietic stem cells was tested for several MPSs. Theoretically, haematopoietic stem cells taken from a normal compatible donor and transplanted into an enzyme-deficient recipient can provide a safe, permanent, and self-replicating source of bone marrow-derived cells. By secreting active lysosomal enzymes, these cells cross-connect nonbone marrow-derived cells. Several animal models for GAGs storage diseases have already been subjected to/undergone BMT. From those experiments, along with human clinical trials already tried, it was possible to verify that there are important variations in therapeutic response among different pathologies with some diseases with CNS pathology which can be successfully treated by BMT (the severe form of MPS I, being the example for the GAG storage disorders; [140]) whereas others cannot (MPS II and III in which BMT was tested with few success; [3, 141]). These variations are usually attributed to the different capacities of secretion, stability, and uptake of each specific enzyme. Nevertheless, important conclusions could be drawn from the collective experience of postnatal transplantation including the idea that the earlier the transplants are performed, the better the clinical response. In the 90s; however, a novel approach started to be tested: ERT. Nowadays, it has been the most tested approach in animal models of GAG storage disorders. Until now, the obtained results have been highlighting the potential of administered recombinant enzyme to reduce GAG accumulation. ERTs are presently available for MPS I (since 2003), II (since 2005), and VI (since 2006). Clinical trials are also in course for MPS IVA treatment through ERT. Nevertheless, this approach is ineffective for the brain since recombinant enzymes are not able to cross the blood-brain barrier (BBB). This is one of the reasons why other therapies are being tested for MPS with CNS involvement. ERT with direct administration of the recombinant enzyme into the brain (intrathecal injections) is also being considered in order to overcome that difficulty. Presently, such approaches are being considered for MPS IIIA and to overcome the cognitive deficit of MPS II and MPS I (reviewed in [142]).

Somatic cell gene transfer is another possible approach, but a long way needs yet to be travelled towards such a therapy is applicable to patients. 

Finally, substrate deprivation therapy (or substrate reduction therapy) is also being considered for some MPSs. This approach is being tested with both genistein and rhodamine B (reviewed in [70]). Genistein, a chemical from the group of isoflavones, has been shown to inhibit the synthesis of GAGs in fibroblasts of patients with various forms of MPSs, namely, types I, II, IIIA, and IIIB [4, 143]. Similar results were obtained with rhodamine B, an inhibitor with an unknown mechanism of action. Remarkably, in MPS IIIA mice treated with rhodamine B, GAG storage decreased not only in somatic tissues, but also in brain, with improved behaviours of the animals [144, 145]. These encouraging results lead to the development of open-label pilot clinical studies with children suffering from Sanfilippo's syndrome types A and B in which a genistein-rich isoflavone extract (SE-2000, Biofarm, Poland) orally administered for 12 months. After one year of treatment, statistically significant improvement in all tested parameters was demonstrated (reviewed in [70]).

In order to better quantify and assess the efficacy of these therapeutic approaches, investigators have been trying to indentify suitable biomarkers for MPS, which allow the evaluation of short- and long-term treatment effects. This is also assuming particular importance since early detection of MPS is an important factor in treatment success. Recently discovered biomarkers include heparin cofactor II-thrombin complex and dermatan sulphate:chondroitin sulphate ratio [146]. Other biomarkers and/or therapeutic targets for MPS joint and bone disease recently identified through animal studies include several proinflammatory cytokines, nitric oxide, and matrix metalloproteinases (MMPs; [147]).

Another hot topic which is recently being discussed refers to the possibility of including some MPSs (particularly type I, IIIA, IIIB, and VI) in neonatal screening programs [148]. The ongoing development of enzyme replacement therapy and other treatments for several LSDs, including MPSs combined with the growing evidence that early commencement of therapy improves outcomes, has increased the pressure for the introduction of newborn screening programs, and a number of pilot studies are ongoing [148–152]. This is only possible thanks to the significant advances that were made in last decade since dried blood spot technology was introduced for enzymatic assays and lysosomal protein profile was developed.

Overall, there are encouraging results, with some therapeutic approaches already approved and others under development. Either way, it is important to stress that the management of MPS requires lifelong attention to the multisystemic involvement by a team of specialists experienced in dealing with these diseases, since none of the therapeutic options currently available result in complete resolution of morbidity.

## Figures and Tables

**Table 1:** Summary table of mucopolysaccharidoses.

Pathology	Subtype	Enzyme deficiency	Gene (localization)	Affected GAG	Clinical manifestations	Animal model
MPS I	Hurler (H)	*α*-L-iduronidase		Dermatan and heparan sulfate	Corneal clouding; dysostosis multiplex; organomegaly; heart disease; mental retardation; death in childhood.	Feline [5]; canine [6]; knock-out mouse [7]
	Hurler -Scheie (H/S)	*α*-L-iduronidase	*IDUA * 4p16.3	Dermatan and heparan sulfate	Intermediate phenotype, between MPS IH and MPS IS.	
	Scheie (S)	*α*-L-iduronidase		Dermatan and heparan sulfate	Corneal clouding; stiff joints; normal intelligence and life span.	
MPS II	Hunter	Irudonate sulfatase	*IDS * Xq28	Dermatan and heparan sulfate	Dysostosis multiplex; organomegaly; no corneal clouding; mental retardation; death before 15 years (severe); Short stature; normal intelligence; survival to 20s to 60s (mild)	Canine [8]; knock-out mouse [9]
MPS III	Sanfilippo A	Heparan-N-sulfatase	*SGSH * 17q25.3	Heparan sulfate	Relatively mild somatic manifestations; hyperactivity; profound mental deterioration.	Canine [10]; spontaneous mouse mutant [11]
	Sanfilippo B	*α*-N-Acetylglucosaminidase	*NAGLU * 17q21	Heparan sulfate	Phenotype similar to MPS IIIA.	Canine [12]
	Sanfilippo C	Heparan acetyl-CoA:*α*-glucosaminide N-acetyltransferase	*HGSNAT* 8p11.1	Heparan sulfate	Phenotype similar to MPS IIIA.	
	Sanfilippo D	N-Acetylglucosamine 6-sulfatase	*GNS * 12q14	Heparan sulfate	Phenotype similar to MPS IIIA.	Caprine [13]
MPS IV	Morquio A	Galactose 6-sulfatase	*GALNS * 16q24.3	Keratan and chondroitin sulfate	Distinctive skeletal abnormalities; corneal clouding; odontoid hypoplasia; milder forms known to exist.	Mouse [14]
	Morquio B	*β*-galactosidase	*GLB1 * 3p21.33	Keratan sulfate	Phenotype similar to MPS IVA, with the same spectrum of severity.	
MPS V	*This designation is no longer used; the phenotype, which was first classified as MPS V, was found to be the milder form of MPS I (Scheie syndrome)*					
MPS VI (Maroteaux-Lamy)		Arylsulfatase B (N-acetylglucosamine 4-sulfatase)	*ARSB * 5q11-q13	dermatan sulfate	Dysostosis multiplex; corneal clouding; normal intelligence; survival to teens in severe form; milder forms also documented.	Feline [15]
MPS VII (Sly)		*β*-glucuronidase	*GUSB * 7q21.11	dermatan, keratan and chondroitin sulfate	Dysostosis multiplex; hepatomegaly; wide spectrum of severity including fetal hydrops and neonatal form.	Canine [16]; spontaneous mouse mutant [17]; mouse [18]
MPS VIII	*∗The designation MPS VIII was based on incorrect data and is no longer used.∗*					
MPS IX		Hyaluronidase 1	*HYAL * 3p21.3			Mouse [19]

**Table 2:** Available therapeutic approaches for mucopolysaccharidoses.

Pathology	Subtype	Available therapeutic approaches
MPS I	Hurler (H)	HSCT (recommended before 2 years of age)
	Hurler -Scheie (H/S)	ERT with Aldurazyme (laronidase; recombinant human *α*-L-iduronidase)
	Scheie (S)	ERT with Aldurazyme (laronidase; recombinant human *α*-L-iduronidase)
MPS II	Hunter	ERT with Elaprase (idursulfase; recombinant human iduronate sulfatase)
MPS III	Sanfilippo A	*Not available*
	Sanfilippo B	*Not available*
	Sanfilippo C	*Not available*
	Sanfilippo D	*Not available*
MPS IV	Morquio A	*ERT: ongoing clinical trial (with recombinant human GALNS)*
	Morquio B	*Not available*
MPS VI (Maroteaux-Lamy)		ERT with Naglazyme (galsulfase; recombinant human arylsulfatase B)
MPS VII (Sly)		*Not available*
MPS IX		*Not available*

## References

[1] Neufeld EF, Muenzer J, Scriver CR, Beaudet AL, Sly WS, Valle D (2001). The mucopolysaccharidoses. *The Metabolic & Molecular Bases of Inherited Disease*.

[2] Giugliani R, Carvalho CG, Herber S, de Camargo Pinto LL (2011). Recent advances in treatment approaches of mucopolysaccharidosis VI. *Current Pharmaceutical Biotechnology*.

[3] Haskins M, Casal M, Ellinwood NM, Melniczek J, Mazrier H, Giger U (2002). Animal models for mucopolysaccharidoses and their clinical relevance. *Acta Paediatrica*.

[4] Piotrowska E, Jakóbkiewicz-Banecka J, Barańska S (2006). Genistein-mediated inhibition of glycosaminoglycan synthesis as a basis for gene expression-targeted isoflavone therapy for mucopolysaccharidoses. *European Journal of Human Genetics*.

[5] Haskins ME, Jezyk PF, Desnick RJ, McDonaugh SK, Patterson DF (1979). Alpha L iduronidase deficiency in a cat: a model of mucopolysaccharidosis I. *Pediatric Research*.

[6] Shull RM, Munger RJ, Spellacy E (1982). Canine *α*-L-iduronidase deficiency. A model of mucopolysaccharidosis I. *American Journal of Pathology*.

[7] Clarke LA, Russell CS, Pownall S (1997). Murine mucopolysaccharidosis type I: targeted disruption of the murine *α*-L-iduronidase gene. *Human Molecular Genetics*.

[8] Wilkerson MJ, Lewis DC, Marks SL, Prieur DJ (1998). Clinical and morphologic features of mucopolysaccharidosis type II in a dog: naturally occurring model of Hunter syndrome. *Veterinary Pathology*.

[9] Garcia AR, Pan J, Lamsa JC, Muenzer J (2007). The characterization of a murine model of mucopolysaccharidosis II (Hunter syndrome). *Journal of Inherited Metabolic Disease*.

[10] Fischer A, Carmichael KP, Munnell JF (1998). Sulfamidase deficiency in a family of dachshunds: a canine model of mucopolysaccharidosis IIIA (Sanfilippo A). *Pediatric Research*.

[11] Bhattacharyya R, Gliddon B, Beccari T, Hopwood JJ, Stanley P (2001). A novel missense mutation in lysosomal sulfamidase is the basis of MPS III A in a spontaneous mouse mutant. *Glycobiology*.

[12] Ellinwood NM, Wang P, Skeen T (2003). A model of mucopolysaccharidosis IIIB (Sanfilippo syndrome type IIIB): N-acetyl-*α*-D-glucosaminidase deficiency in Schipperke dogs. *Journal of Inherited Metabolic Disease*.

[13] Thompson JN, Jones MZ, Dawson G, Huffman PS (1992). N-acetylglucosamine 6-sulphatase deficiency in a Nubian goat: a model of Sanfilippo syndrome type D (mucopoly-saccharidosis IIID). *Journal of Inherited Metabolic Disease*.

[14] Tomatsu S, Orii KO, Vogler C (2003). Mouse model on N-acetylgalactosamine-6-sulfate sulfatase deficiency (Galns-/-) produced by targeted disruption of the gene defective in Morquio A disease. *Human Molecular Genetics*.

[15] MacRì B, Marino F, Mazzullo G (2002). Mucopolysaccharidosis VI in a Siamese/short-haired European cat. *Journal of Veterinary Medicine A*.

[16] Haskins ME, Desnick RJ, DiFerrante N (1984). *β*-glucuronidase deficiency in a dog: a model of human mucopolysaccharidosis VII. *Pediatric Research*.

[17] Birkenmeier EH, Davisson MT, Beamer WG (1989). Murine mucopolysaccharidosis type VII. Characterization of a mouse with *β*-glucuronidase deficiency. *Journal of Clinical Investigation*.

[18] Tomatsu S, Sukegawa K, Trandafirescu GG (2006). Differences in methylation patterns in the methylation boundary region of IDS gene in hunter syndrome patients: implications for CpG hot spot mutations. *European Journal of Human Genetics*.

[19] Martin DC, Atmuri V, Hemming RJ (2008). A mouse model of human mucopolysaccharidosis IX exhibits osteoarthritis. *Human Molecular Genetics*.

[20] McKusick VA (1972). *Heritable Disorders of Connective Tissue*.

[21] Scott HS, Guo XH, Hopwood JJ, Morris CP (1992). Structure and sequence of the human *α*-L-iduronidase gene. *Genomics*.

[22] Scott HS, Ashton LJ, Eyre HJ (1990). Chromosomal localization of the human *α*-L-iduronidase gene (IDUA) to 4p16.3. *American Journal of Human Genetics*.

[23] Spellacy E, Shull RM, Constantopoulos G, Neufeld EF (1983). A canine model of human *α*-L-iduronidase deficiency. *Proceedings of the National Academy of Sciences of the United States of America*.

[24] Stoltzfus LJ, Sosa-Pineda B, Moskowitz SM (1992). Cloning and characterization of cDNA encoding canine *α*-L-iduronidase. mRNA deficiency in mucopolysaccharidosis I dog. *Journal of Biological Chemistry*.

[25] Menon KP, Tieu PT, Neufeld EF (1992). Architecture of the canine IDUA gene and mutation underlying canine mucopolysaccharidosis I. *Genomics*.

[26] Shull RM, Kakkis ED, McEntee MF, Kania SA, Jonas AJ, Neufeld EF (1994). Enzyme replacement in a canine model of Hurler syndrome. *Proceedings of the National Academy of Sciences of the United States of America*.

[27] Grosson CLS, MacDonald ME, Duyao MP, Ambrose CM, Roffler Tarlov S, Gusella JF (1994). Synteny conservation of the Huntington’s disease gene and surrounding loci on mouse chromosome 5. *Mammalian Genome*.

[28] Russell C, Hendson G, Jevon G (1998). Murine MPS I: insights into the pathogenesis of Hurler syndrome. *Clinical Genetics*.

[29] Peters C, Shapiro EG, Krivit W (1998). Neuropsychological development in children with Hurler syndrome following hematopoietic stem cell transplantation. *Pediatric Transplantation*.

[30] Peters C, Shapiro EG, Krivit W (1998). Hurler syndrome: past, present, and future. *Journal of Pediatrics*.

[31] Staba SL, Escolar ML, Poe M (2004). Cord-blood transplants from unrelated donors in patients with Hurler’s syndrome. *New England Journal of Medicine*.

[32] Boelens JJ, Wynn RF, O’Meara A (2007). Outcomes of hematopoietic stem cell transplantation for Hurler’s syndrome in Europe: a risk factor analysis for graft failure. *Bone Marrow Transplantation*.

[33] Clarke LA, Wraith JE, Beck M (2009). Long-term efficacy and safety of laronidase in the treatment of mucopolysaccharidosis I. *Pediatrics*.

[34] Wraith JE, Clarke LA, Beck M (2004). Enzyme replacement therapy for mucopolysaccharidosis I: a randomized, double-blinded, placebo-controlled, multinational study of recombinant human *α*-L-iduronidase (laronidase). *Journal of Pediatrics*.

[35] Harada H, Uchiwa H, Nakamura M (2011). Laronidase replacement therapy improves myocardial function in mucopolysaccharidosis I. *Molecular Genetics and Metabolism*.

[36] Tylki-Szymanska A, Marucha J, Jurecka A, Syczewska M, Czartoryska B (2010). Efficacy of recombinant human *α*-L-iduronidase (laronidase) on restricted range of motion of upper extremities in mucopolysaccharidosis type i patients. *Journal of Inherited Metabolic Disease*.

[37] Tylki-Szymanska A, Rozdzynska A, Jurecka A, Marucha J, Czartoryska B (2010). Anthropometric data of 14 patients with mucopolysaccharidosis I: retrospective analysis and efficacy of recombinant human *α*-l-iduronidase (laronidase). *Molecular Genetics and Metabolism*.

[38] Gabrielli O, Clarke LA, Bruni S, Coppa GV (2010). Enzyme-replacement therapy in a 5-month-old boy with attenuated presymptomatic MPS I: 5-year follow-up. *Pediatrics*.

[39] Wraith JE, Rogers JG, Danks DM (1987). The mucopolysaccharidoses. *Australian Paediatric Journal*.

[40] Gorlin RJ, Cohen MM, Hennekam RCM (2001). *Syndromes of the Head and Neck*.

[41] Donaldson MDC, Pennock CA, Berry PJ, Duncan AW, Cawdery JE, Leonard JV (1989). Hurler syndrome with cardiomyopathy in infancy. *Journal of Pediatrics*.

[42] Shapiro J, Strome M, Crocker AC (1985). Airway obstruction and sleep apnea in Hurler and Hunter syndromes. *Annals of Otology, Rhinology and Laryngology*.

[43] Hanson M, Lupski JR, Hicks J, Metry D (2003). Association of dermal melanocytosis with lysosomal storage disease: clinical features and hypotheses regarding pathogenesis. *Archives of Dermatology*.

[44] McKusick VA, Kaplan D, Wise D (1965). The genetic mucopolysaccharidoses. *Medicine*.

[45] Whitley CB, Beighton P (1993). The mucopolysaccharidoses. *McKusick's Heritable Disorders of Connective Tissue*.

[46] Perks WH, Cooper RA, Bradbury S (1980). Sleep apnoea in Scheie's syndrome. *Thorax*.

[47] Wraith JE, Scarpa M, Beck M (2008). Mucopolysaccharidosis type II (Hunter syndrome): a clinical review and recommendations for treatment in the era of enzyme replacement therapy. *European Journal of Pediatrics*.

[48] Wilson PJ, Suthers GK, Callen DF (1991). Frequent deletions at Xq28 indicate genetic heterogeneity in Hunter syndrome. *Human Genetics*.

[49] Flomen RH, Green EP, Green PM, Bentley DR, Giannelli F (1993). Determination of the organisation of coding sequences within the iduronate sulphate sulphatase (IDS) gene. *Human Molecular Genetics*.

[50] Wilson PJ, Meaney CA, Hopwood JJ, Morris CP (1993). Sequence of the human iduronate 2-sulfatase (IDS) gene. *Genomics*.

[51] Bondeson ML, Malmgren H, Dahl N, Carlberg BM, Pettersson U (1995). Presence of an IDS-related locus (IDS2) and Xq28 complicates the mutational analysis of Hunter syndrome. *European Journal of Human Genetics*.

[52] Peters C, Balthazor M, Shapiro EG (1996). Outcome of unrelated donor bone marrow transplantation in 40 children with Hurler syndrome. *Blood*.

[53] Coppa GV, Gabrielli O, Zampini L (1995). Bone marrow transplantation in Hunter syndrome (mucopolysaccharidosis type II): two year follow-up of the first Italian patient and review of the literature. *Pediatria Medica e Chirurgica*.

[54] McKinnis EJR, Sulzbacher S, Rutledge JC, Sanders J, Scott CR (1996). Bone marrow transplantation in Hunter syndrome. *Journal of Pediatrics*.

[55] Burrow TA, Leslie ND (2008). Review of the use of idursulfase in the treatment of mucopolysaccharidosis II. *Biologics*.

[56] Muenzer J, Lamsa JC, Garcia A, Dacosta J, Garcia J, Treco DA (2002). Enzyme replacement therapy in mucopolysaccharidosis type II (Hunter syndrome): a preliminary report. *Acta Paediatrica*.

[57] Garcia AR, DaCosta JM, Pan J, Muenzer J, Lamsa JC (2007). Preclinical dose ranging studies for enzyme replacement therapy with idursulfase in a knock-out mouse model of MPS II. *Molecular Genetics and Metabolism*.

[58] Muenzer J, Gucsavas-Calikoglu M, McCandless SE, Schuetz TJ, Kimura A (2007). A phase I/II clinical trial of enzyme replacement therapy in mucopolysaccharidosis II (Hunter syndrome). *Molecular Genetics and Metabolism*.

[59] Muenzer J, Wraith JE, Beck M (2006). A phase II/III clinical study of enzyme replacement therapy with idursulfase in mucopolysaccharidosis II (Hunter syndrome). *Genetics in Medicine*.

[60] Papadia F, Lozupone MS, Gaeta A, Capodiferro D, Lacalendola G (2011). Long-term enzyme replacement therapy in a severe case of mucopolysaccharidosis type II (Hunter syndrome). *European Review for Medical and Pharmacological Sciences*.

[61] Filippon L, Wayhs CAY, Atik DM (2011). DNA damage in leukocytes from pretreatment mucopolysaccharidosis type II patients; protective effect of enzyme replacement therapy. *Mutation Research*.

[62] Filippon L, Vanzin CS, Biancini GB (2011). Oxidative stress in patients with mucopolysaccharidosis type II before and during enzyme replacement therapy. *Molecular Genetics and Metabolism*.

[63] Van De Kamp JJP, Niermeijer MF, Von Figura K, Giesberts MAH (1981). Genetic heterogeneity and clinical variability in the Sanfilippo syndrome (types A, B, and C). *Clinical Genetics*.

[64] Scott HS, Blanch L, Guo XH (1995). Cloning of the sulphamidase gene and identification of mutations in Sanfilippo A syndrome. *Nature Genetics*.

[65] Karageorgos LE, Guo XH, Blanch L (1996). Structure and sequence of the human sulphamidase gene. *DNA Research*.

[66] Aronovich EL, Carmichael KP, Morizono H (2000). Canine heparan sulfate sulfamidase and the molecular pathology underlying Sanfilippo syndrome type A in Dachshunds. *Genomics*.

[67] Hemsley KM, Hopwood JJ (2005). Development of motor deficits in a murine model of mucopolysaccharidosis type IIIA (MPS-IIIA). *Behavioural Brain Research*.

[68] Settembre C, Fraldi A, Jahreiss L (2008). A block of autophagy in lysosomal storage disorders. *Human Molecular Genetics*.

[69] Roberts ALK, Thomas BJ, Wilkinson AS, Fletcher JM, Byers S (2006). Inhibition of glycosaminoglycan synthesis using rhodamine B in a mouse model of mucopolysaccharidosis type IIIA. *Pediatric Research*.

[70] Jakóbkiewicz-Banecka J, Wegrzyn A, Wegrzyn G (2007). Substrate deprivation therapy: a new hope for patients suffering from neuronopathic forms of inherited lysosomal storage diseases. *Journal of Applied Genetics*.

[71] Dziedzic D, Wgrzyn G, Jakóbkiewicz-Banecka J (2010). Impairment of glycosaminoglycan synthesis in mucopolysaccharidosis type IIIA cells by using siRNA: a potential therapeutic approach for Sanfilippo disease. *European Journal of Human Genetics*.

[72] Fraldi A, Hemsley K, Crawley A (2007). Functional correction of CNS lesions in an MPS-IIIA mouse model by intracerebral AAV-mediated delivery of sulfamidase and SUMF1 genes. *Human Molecular Genetics*.

[73] Zhao HG, Li HH, Schmidtchen A, Bach G, Neufeld EF (1995). The gene encoding alpha-N-acetylglucosaminidase and mutations underlying Sanfilippo B syndrome. *American Journal of Human Genetics*.

[74] Zhao HG, Li HH, Bach G, Schmidtchen A, Neufeld EF (1996). The molecular basis of Sanfilippo syndrome type B. *Proceedings of the National Academy of Sciences of the United States of America*.

[75] Li HH, Yu WH, Rozengurt N (1999). Mouse model of Sanfilippo syndrome type B produced by targeted disruption of the gene encoding alpha-N-acetylglucosaminidase. *Proc Natl Acad Sci USA*.

[76] Yu WH, Zhao KW, Ryazantsev S, Rozengurt N, Neufeld EF (2000). Short-term enzyme replacement in the murine model of sanfilippo syndrome type B. *Molecular Genetics and Metabolism*.

[77] Zhao KW, Neufeld EF (2000). Purification and characterization of recombinant human *α*-N-acetylglucosaminidase secreted by Chinese hamster ovary cells. *Protein Expression and Purification*.

[78] Weber B, Hopwood JJ, Yogalingam G (2001). Expression and characterization of human recombinant and *α*-N-actylglucosaminidase. *Protein Expression and Purification*.

[79] Beck M (2007). New therapeutic options for lysosomal storage disorders: enzyme replacement, small molecules and gene therapy. *Human Genetics*.

[80] Fu H, Samulski RJ, McCown TJ, Picornell YJ, Fletcher D, Muenzer J (2002). Neurological correction of lysosomal storage in a mucopolysaccharidosis IIIB mouse model by adeno-associated virus-mediated gene delivery. *Molecular Therapy*.

[81] Cressant A, Desmaris N, Verot L (2004). Improved behavior and neuropathology in the mouse model of sanfilippo type IIIB disease after adeno-associated virus-mediated gene transfer in the striatum. *Journal of Neuroscience*.

[82] Heldermon CD, Ohlemiller KK, Herzog ED (2010). Therapeutic efficacy of bone marrow transplant, intracranial AAV-mediated gene therapy, or both in the mouse model of MPS IIIB. *Molecular Therapy*.

[83] Di Natale P, Di Domenico C, Gargiulo N (2005). Treatment of the mouse model of mucopolysaccharidosis type IIIB with lentiviral-NAGLU vector. *Biochemical Journal*.

[84] Villani GRD, Follenzi A, Vanacore B, Di Domenico C, Naldini L, Di Natale P (2002). Correction of mucopolysaccharidosis type IIIB fibroblasts by lentiviral vector-mediated gene transfer. *Biochemical Journal*.

[85] Di Domenico C, Villani GRD, Di Napoli D (2009). Intracranial gene delivery of LV-NAGLU vector corrects neuropathology in murine MPS IIIB. *American Journal of Medical Genetics A*.

[86] Fan X, Zhang H, Zhang S (2006). Identification of the gene encoding the enzyme deficient in mucopolysaccharidosis IIIC (Sanfilippo disease type C). *American Journal of Human Genetics*.

[87] Hřebíček M, Mrázová L, Seyrantepe V (2006). Mutations in TMEM76* cause mucopolysaccharidosis IIIC (Sanfilippo C syndrome). *American Journal of Human Genetics*.

[88] Feldhammer M, Durand S, Pshezhetsky AV (2009). Protein misfolding as an underlying molecular defect in mucopolysaccharidosis III type C. *PloS one*.

[89] Durand S, Feldhammer M, Bonneil E, Thibault P, Pshezhetsky AV (2010). Analysis of the biogenesis of heparan sulfate acetyl-CoA: *α*- glucosaminide N-acetyltransferase provides insights into the mechanism underlying its complete deficiency in mucopolysaccharidosis IIIC. *Journal of Biological Chemistry*.

[90] Fedele AO, Hopwood JJ (2010). Functional analysis of the HGSNAT gene in patients with mucopolysaccharidosis IIIC (Sanfilippo C syndrome). *Human Mutation*.

[91] Freeman C, Clements PR, Hopwood JJ (1987). Human liver N-acetylglucosamine-6-sulphate sulphatase. Purification and characterization. *Biochemical Journal*.

[92] Freeman C, Hopwood JJ (1987). Human liver N-acetylglucosamine-6-phosphate sulphatase. Catalytic properties. *Biochemical Journal*.

[93] Robertson DA, Callen DF, Baker EG, Morris CP, Hopwood JJ (1988). Chromosomal localization of the gene for human glucosamine-6-sulphatase to 12q14. *Human Genetics*.

[94] Mok A, Cao H, Hegele RA (2003). Genomic basis of mucopolysaccharidosis type IIID (MIM 252940) revealed by sequencing of GNS encoding N-acetylglucosamine-6-sulfatase. *Genomics*.

[95] Beesley CE, Burke D, Jackson M, Vellodi A, Winchester BG, Young EP (2003). Sanfilippo syndrome type D: identification of the first mutation in the N-acetylglucosamine-6-sulphatase gene. *Journal of Medical Genetics*.

[96] Downs-Kelly E, Jones MZ, Alroy J (2000). Caprine mucopolysaccharidosis IIID: a preliminary trial of enzyme replacement therapy. *Journal of Molecular Neuroscience*.

[97] Montaño AM, Tomatsu S, Brusius A, Smith M, Orii T (2008). Growth charts for patients affected with Morquio A disease. *American Journal of Medical Genetics A*.

[98] Tomatsu S, Fukuda S, Masue M, Sukegawa K, Masuno M, Orii T (1992). Mucopolysaccharidosis type IVA: characterization and chromosomal localization of N-acetylgalactosamine-6-sulfate sulfatase gene and genetic heterogeneity. *American Journal of Human Genetics*.

[99] Nakashima Y, Tomatsu S, Hori T (1994). Mucopolysaccharidosis IV A: molecular cloning of the human N- acetylgalactosamine-6-sulfatase gene (GALNS) and analysis of the 5’-flanking region. *Genomics*.

[100] Morris CP, Guo XH, Apostolou S, Hopwood JJ, Scott HS (1994). Morquio A syndrome: cloning, sequence, and structure of the human N- acetylgalactosamine 6-sulfatase (GALNS) gene. *Genomics*.

[101] Tomatsu S, Montaño AM, Ohashi A (2008). Enzyme replacement therapy in a murine model of Morquio A syndrome. *Human Molecular Genetics*.

[102] Tomatsu S, Montaño AM, Oikawa H (2011). Mucopolysaccharidosis type IVA (morquio a disease): clinical review and current treatment: a special review. *Current Pharmaceutical Biotechnology*.

[103] Rodríguez A, Espejo AJ, Hernández A (2010). Enzyme replacement therapy for Morquio A: an active recombinant N-acetylgalactosamine-6-sulfate sulfatase produced in Escherichia coli BL21. *Journal of Industrial Microbiology and Biotechnology*.

[104] Oshima A, Tsuji A, Nagao Y, Sakuraba H, Suzuki Y (1988). Cloning, sequencing, and expression of cDNA for human *β*-galactosidase. *Biochemical and Biophysical Research Communications*.

[105] Santamaria R, Blanco M, Chabas A, Grinberg D, Vilageliu L (2007). Identification of 14 novel GLB1 mutations, including five deletions, in 19 patients with GM1 gangliosidosis from South America. *Clinical Genetics*.

[106] Takano T, Yamanouchi Y (1993). Assignment of human *β*-galactosidase-A gene to 3p21.33 by fluorescence in situ hybridization. *Human Genetics*.

[107] Morreau H, Galjart NJ, Gillemans N, Willemsen R, Van Der Horst GTJ, D’Azzo A (1989). Alternative splicing of *β*-galactosidase mRNA generates the classic lysosomal enzyme and a *β*-galactosidase-related protein. *Journal of Biological Chemistry*.

[108] Hinek E (1996). Biological roles of the non-integrin elastin/laminin receptor. *Biological Chemistry*.

[109] Privitera S, Prody CA, Callahan JW, Hinek A (1998). The 67-kDa enzymatically inactive alternatively spliced variant of *β*- galactosidase is identical to the elastin/laminin-binding protein. *Journal of Biological Chemistry*.

[110] Azevedo ACMM, Schwartz IV, Kalakun L (2004). Clinical and biochemical study of 28 patients with mucopolysaccharides type VI. *Clinical Genetics*.

[111] Karageorgos L, Brooks DA, Pollard A (2007). Mutational analysis of 105 mucopolysaccharidosis type VI patients. *Human Mutation*.

[112] Fidzianska E, Abramowicz T, Czartoryska B (1984). Assignment of the gene for human arylsulfatase B, ARSB, to chromosome region 5p11 → 5qter. *Cytogenetics and Cell Genetics*.

[113] Auclair D, Hopwood JJ, Brooks DA, Lemontt JF, Crawley AC (2003). Replacement therapy in Mucopolysaccharidosis type VI: advantages of early onset of therapy. *Molecular Genetics and Metabolism*.

[114] Auclair D, Finnie J, White J (2010). Repeated intrathecal injections of recombinant human 4-sulphatase remove dural storage in mature mucopolysaccharidosis VI cats primed with a short-course tolerisation regimen. *Molecular Genetics and Metabolism*.

[115] Auclair D, Hopwood JJ, Lemontt JF, Chen L, Byers S (2007). Long-term intra-articular administration of recombinant human N-acetylgalactosamine-4-sulfatase in feline mucopolysaccharidosis VI. *Molecular Genetics and Metabolism*.

[116] Harmatz P, Giugliani R, D. Schwartz IV (2008). Long-term follow-up of endurance and safety outcomes during enzyme replacement therapy for mucopolysaccharidosis VI: final results of three clinical studies of recombinant human N-acetylgalactosamine 4-sulfatase. *Molecular Genetics and Metabolism*.

[117] Byers S, Rothe M, Lalic J, Koldej R, Anson DS (2009). Lentiviral-mediated correction of MPS VI cells and gene transfer to joint tissues. *Molecular Genetics and Metabolism*.

[118] Cotugno G, Annunziata P, Tessitore A (2011). Long-term amelioration of feline mucopolysaccharidosis VI after AAV-mediated liver gene transfer. *Molecular Therapy*.

[119] Shipley JM, Klinkenberg M, Wu BM, Bachinsky DR, Grubb JH, Sly WS (1993). Mutational analysis of a patient with mucopolysaccharidosis type VII, and identification of pseudogenes. *American Journal of Human Genetics*.

[120] Sly WS, Quinton BA, McAlister WH, Rimoin DL (1973). Beta glucuronidase deficiency: report of clinical, radiologic, and biochemical features of a new mucopolysaccharidosis. *The Journal of Pediatrics*.

[121] Miller RD, Hoffmann JW, Powell PP (1990). Cloning and characterization of the human *β*-glucuronidase gene. *Genomics*.

[122] Speleman F, Vervoort R, Van Roy N, Liebaers I, Sly WS, Lissens W (1996). Localization by fluorescence in situ hybridization of the human functional *β*-glucuronidase gene (GUSB) to 7q11.21 → q11.22 and two pseudogenes to 5p13 and 5q13. *Cytogenetics and Cell Genetics*.

[123] Shipley JM, Klinkenberg M, Wu BM, Bachinsky DR, Grubb JH, Sly WS (1993). Mutational analysis of a patient with mucopolysaccharidosis type VII, and identification of pseudogenes. *American Journal of Human Genetics*.

[124] Tomatsu S, Montano AM, Dung VC, Grubb JH, Sly WS (2009). Mutations and polymorphisms in GUSB gene in mucopolysaccharidosis VII (sly syndrome). *Human Mutation*.

[125] Wu BM, Tomatsu S, Fukuda S, Sukegawa K, Orii T, Sly WS (1994). Overexpression rescues the mutant phenotype of L176F mutation causing *β*- glucuronidase deficiency mucopolysaccharidosis in two Mennonite siblings. *Journal of Biological Chemistry*.

[126] Vervoort R, Islam MR, Sly WS (1996). Molecular analysis of patients with *β*-glucuronidase deficiency presenting as hydrops fetalis or as early mucopolysaccharidosis VII. *American Journal of Human Genetics*.

[127] Schwartz I, Silva LR, Leistner S (2003). Mucopolysaccharidosis VII: clinical, biochemical and molecular investigation of a Brazilian family. *Clinical Genetics*.

[128] Haskins ME, Aguirre GD, Jezyk PF, Schuchman EH, Desnick RJ, Patterson DF (1991). Mucopolysaccharidosis type VII (Sly syndrome): beta-glucuronidase-deficient mucopolysaccharidosis in the dog. *American Journal of Pathology*.

[129] Dombrowski DCS, Carmichael KP, Wang P, O’Malley TM, Haskins ME, Giger U (2004). Mucopolysaccharidosis type VII in a German shepherd dog. *Journal of the American Veterinary Medical Association*.

[130] Bosch A, Perret E, Desmaris N, Heard JM (2000). Long-term and significant correction of brain lesions in adult mucopolysaccharidosis type VII mice using recombinant AAV vectors. *Molecular Therapy*.

[131] Bosch A, Perret E, Desmaris N, Trono D, Heard JM (2000). Reversal of pathology in the entire brain of mucopolysaccharidosis type VII mice after lentivirus-mediated gene transfer. *Human Gene Therapy*.

[132] Yamada Y, Kato K, Sukegawa K (1998). Treatment of MPS VII (Sly disease) by allogeneic BMT in a female with homozygous A619V mutation. *Bone Marrow Transplantation*.

[133] Ginsburg LC, DiFerrante DT, Caskey CT, DiFerrante NM (1977). Glucosamine-6-SO_4_ sulfatase deficiency: a new mucopolysaccharidosis. *Clinical Research*.

[134] Ginsberg LC, Donnelly PV, Di Ferrante DT (1978). N-acetylglucosamine-6-sulfate sulfatase in man: deficiency of the enzyme in a new mucopolysaccharidosis. *Pediatric Research*.

[135] Di Ferrante N (1980). N-acetylglucosamine-6-sulfate sulfatase deficiency reconsidered. *Science*.

[136] Natowicz MR, Short MP, Wang Y (1996). Brief report: clinical and biochemical manifestations of hyaluronidase deficiency. *New England Journal of Medicine*.

[137] Triggs-Raine B, Salo TJ, Zhang H, Wicklow BA, Natowicz MR (1999). Mutations in HYAL1, a member of a tandemly distributed multigene family encoding disparate hyaluronidase activities, cause a newly described lysosomal disorder, mucopolysaccharidosis IX. *Proceedings of the National Academy of Sciences of the United States of America*.

[138] Csoka AB, Frost GI, Stern R (2001). The six hyaluronidase-like genes in the human and mouse genomes. *Matrix Biology*.

[139] Jadin L, Wu X, Ding H (2008). Skeletal and hematological anomalies in HYAL2-deficient mice: a second type of mucopolysaccharidosis IX?. *FASEB Journal*.

[140] Krivit W (2004). Allogeneic stem cell transplantation for the treatment of lysosomal and peroxisomal metabolic diseases. *Springer Seminars in Immunopathology*.

[141] Lachmann R (2010). Treatments for lysosomal storage disorders. *Biochemical Society Transactions*.

[142] Dickson PI, Chen AH (2011). Intrathecal enzyme replacement therapy for mucopolysaccharidosis I: translating success in animal models to patients. *Current Pharmaceutical Biotechnology*.

[143] Friso A, Tomanin R, Salvalaio M, Scarpa M (2010). Genistein reduces glycosaminoglycan levels in a mouse model of mucopolysaccharidosis type II. *British Journal of Pharmacology*.

[144] Roberts ALK, Thomas BJ, Wilkinson AS, Fletcher JM, Byers S (2006). Inhibition of glycosaminoglycan synthesis using rhodamine B in a mouse model of mucopolysaccharidosis type IIIA. *Pediatric Research*.

[145] Roberts ALK, Rees MH, Klebe S, Fletcher JM, Byers S (2007). Improvement in behaviour after substrate deprivation therapy with rhodamine B in a mouse model of MPS IIIA. *Molecular Genetics and Metabolism*.

[146] Langford-Smith KJ, Mercer J, Petty J (2011). Heparin cofactor II-thrombin complex and dermatan sulphate: chondroitin sulphate ratio are biomarkers of short- and long-term treatment effects in mucopolysaccharide diseases. *Journal of Inherited Metabolic Disease*.

[147] Simonaro CM, D’Angelo M, Haskins ME, Schuchman EH (2005). Joint and bone disease in mucopolysaccharidoses VI and VII: identification of new therapeutic targets and BioMarkers using animal models. *Pediatric Research*.

[148] Fuller M, Tucker JN, Lang DL (2011). Screening patients referred to a metabolic clinic for lysosomal storage disorders. *Journal of Medical Genetics*.

[149] Spada M, Pagliardini S, Yasuda M (2006). High incidence of later-onset Fabry disease revealed by newborn screening. *American Journal of Human Genetics*.

[150] Lin HY, Chong KW, Hsu JH (2009). High incidence of the cardiac variant of fabry disease revealed by newborn screening in the Taiwan Chinese population. *Circulation*.

[151] Chien YH, Chiang SC, Zhang XK (2008). Early detection of pompe disease by newborn screening is feasible: results from the Taiwan screening program. *Pediatrics*.

[152] Duffner PK, Caggana M, Orsini JJ (2009). Newborn screening for Krabbe disease: the New York state model. *Pediatric Neurology*.

